# Correlation of abnormal brain changes with perinatal factors in very preterm infants based on diffusion tensor imaging

**DOI:** 10.3389/fnins.2023.1137559

**Published:** 2023-03-30

**Authors:** Ying Liu, Binbin Nie, Yituo Wang, Fang He, Qiaozhi Ma, Tao Han, Guangjuan Mao, Jiqiang Liu, Haiping Zu, Xuetao Mu, Bing Wu

**Affiliations:** ^1^School of Medical Imaging, Weifang Medical University, Weifang, Shandong, China; ^2^Department of Radiology, The Third Medical Center of Chinese PLA General Hospital, Beijing, China; ^3^Beijing Engineering Research Center of Radiographic Techniques and Equipment, Institute of High Energy Physics, Chinese Academy of Sciences, Beijing, China; ^4^Department of Radiology, Seventh Medical Center of Chinese PLA General Hospital, Beijing, China; ^5^Child Growth and Development Clinic, Seventh Medical Center of Chinese PLA General Hospital, Beijing, China; ^6^Department of Neonatology, Seventh Medical Center of Chinese PLA General Hospital, Beijing, China; ^7^School of Nuclear Science and Technology, University of Chinese Academy of Sciences, Beijing, China; ^8^Department of Magnetic Resonance Imaging, The Third Affiliated Hospital of Xinxiang Medical University, Xinxiang, Henan, China; ^9^Department of Radiology, Specialized Medical Center of the Rocket Army, Beijing, China

**Keywords:** preterm infants, diffusion tensor imaging, perinatal factors, connectivity networks, tractography

## Abstract

**Background:**

It remains unclear whether very preterm (VP) infants have the same level of brain structure and function as full-term (FT) infants. In addition, the relationship between potential differences in brain white matter microstructure and network connectivity and specific perinatal factors has not been well characterized.

**Objective:**

This study aimed to investigate the existence of potential differences in brain white matter microstructure and network connectivity between VP and FT infants at term-equivalent age (TEA) and examine the potential association of these differences with perinatal factors.

**Methods:**

A total of 83 infants were prospectively selected for this study: 43 VP infants (gestational age, or GA: 27–32 weeks) and 40 FT infants (GA: 37–44 weeks). All infants at TEA underwent both conventional magnetic resonance imaging (MRI) and diffusion tensor imaging (DTI). Significant differences in white matter fractional anisotropy (FA) and mean diffusivity (MD) images between the VP and FT groups were observed using tract-based spatial statistics (TBSS). The fibers were tracked between each pair of regions in the individual space, using the automated anatomical labeling (AAL) atlas. Then, a structural brain network was constructed, where the connection between each pair of nodes was defined by the number of fibers. Network-based statistics (NBS) were used to examine differences in brain network connectivity between the VP and FT groups. Additionally, multivariate linear regression was conducted to investigate potential correlations between fiber bundle numbers and network metrics (global efficiency, local efficiency, and small-worldness) and perinatal factors.

**Results:**

Significant differences in FA were observed between the VP and FT groups in several regions. These differences were found to be significantly associated with perinatal factors such as bronchopulmonary dysplasia (BPD), activity, pulse, grimace, appearance, respiratory (APGAR) score, gestational hypertension, and infection. Significant differences in network connectivity were observed between the VP and FT groups. Linear regression results showed significant correlations between maternal years of education, weight, the APGAR score, GA at birth, and network metrics in the VP group.

**Conclusions:**

The findings of this study shed light on the influence of perinatal factors on brain development in VP infants. These results may serve as a basis for clinical intervention and treatment to improve the outcome of preterm infants.

## 1. Introduction

Preterm birth is a crucial global health issue. It has been estimated that preterm birth accounts for ~10% of all births worldwide (Chawanpaiboon et al., 2019). The implementation of China's two-child policy has increased the country's overall birth rate (Liu et al., 2020). However, many perinatal factors, such as advanced maternal age, maternal complications, and multiple pregnancies, increased the overall preterm birth rate in China from 5.9% in 2012 to 6.4% in 2018 (Deng et al., 2021). Premature delivery, to varying degrees, disrupts the sequence of the neural and anatomical development processes of the fetus in the mother. Not only does premature delivery affect white matter tracts, but it also greatly affects the structural connectivity of the brain. In addition, perinatal factors can also contribute to the occurrence of preterm birth and alter brain development in preterm infants. Perinatal factors refer to the important period before and after birth, from 28 weeks of pregnancy to 1 week after delivery. There are three stages: prenatal factors, intrapartum factors, and postnatal factors. They cause brain development in fetuses or infants at different stages.

Diffusion tensor imaging (DTI) is a widely used technique to explore the developmental trajectory of the infant brain (Pecheva et al., 2018). It measures the degree of anisotropy within a given voxel, which is an important indicator of the degree of myelination or the integrity of the fiber tracts. DTI-derived indicators, such as increasing fractional anisotropy (FA) and decreasing mean diffusivity (MD), typically observed with increasing gestational age (GA) at birth, play a crucial role in the study of brain development in infants. Tracer-based spatial statistics (TBSS) (Smith et al., 2006) allow the detection of changes in white matter microstructure in preterm infants without focal brain injury. In addition to white matter tracts, there is growing evidence that (van den Heuvel et al., 2015; Batalle et al., 2017; He et al., 2018) alterations in an infant's brain network connectome can predict brain injury and neurodevelopmental outcomes. Based on graph theory, the two main aspects of a graph theory-based human brain connectome are the structural network and the functional network. The functional network is established by the coherence of brain functional fluctuations, while the structural network is established by the fiber bundle connections (Grayson and Fair, 2017; Keunen et al., 2017). Graph theory-based structural networks are used to assess white matter tract networks. In contrast, the construction of structural networks in the human brain is mainly based on structural and diffusion magnetic resonance imaging (MRI) images, and the nodes and edges of the network are used to construct a network matrix. Nodes represent different brain regions, including cortical and subcortical gray matter structures. Edges are the connections between each pair of nodes defined by fiber bundle numbers (Rubinov and Sporns, 2010). Four network metrics, such as global efficiency, local efficiency, fiber bundle numbers, and small-worldness, are generally used to describe graph theory-based structural networks. These networks allow for the visualization of structural brain connections and a more intuitive analysis of the change in structural brain connections in relevant brain regions.

There is much research on brain structure and dysfunction in preterm infants worldwide; however, there is little research on the existence of abnormal white matter tracts and structural networks in very preterm (VP) infants with normal MRI at term-equivalent age (TEA). There is a paucity of research on the correlation between perinatal factors and abnormalities in preterm infants in China. Assuming that there were still differences in brain fiber bundles and structural networks between VP and full-term (FT) infants at TEA and that these differences were correlated with China-specific perinatal factors, TBSS and graph theory-based structural networks were proposed in this study to study brain fiber tracts and networks in VP infants and also the effects of perinatal factors on the structure of white matter tracts and networks to provide a theoretical basis for early intervention and treatment of VP infants.

## 2. Materials and methods

### 2.1. Participants

During a period from July 2021 to November 2022, a total of 120 infants, 65 VP infants, and 55 control FT infants were recruited into this study and underwent MRI brain scans at the TEA. Parents of all participants provided written informed consent, and ethical approval was obtained from the Institutional Review Board of the local hospital. The inclusion criteria for the VP group included: (1) VP infants born at 28–32 weeks of GA and postmenstrual age (PMA) of <43 weeks at the time of an MRI scan; (2) no evidence of congenital infection or multiple congenital anomaly syndrome; and (3) normal brain MRI [T1-weighted (T1w) imaging, T2-weighted imaging, T2-fluid attenuated inversion recovery (T2-FLAIR), and diffusion-weighted imaging (DWI)]. The exclusion criteria included (1) preterm infants at 33–36 weeks GA; (2) PMA of >43 weeks at the time of an MRI scan; (3) any acquired lesions on MRI (assessed by a radiologist); (4) visible artifacts on MRI; (5) congenital malformations or syndromes; and (6) encephalopathy due to various factors. The control group included healthy FT infants (≥37 weeks GA at birth and PMA: 37–42 weeks).

### 2.2. Perinatal factors

Perinatal data on 120 infants were collected from a medical record review. We selected prenatal factors including maternal age at delivery, mode of delivery, gestational hypertension, gestational diabetes, time to premature rupture of the fetal membranes (>18 h), maternal fever, intrauterine infection, and maternal education. Intrapartum factors such as GA, sex, weight, activity, pulse, grimace, appearance, respiration (APGAR) score (a 5-min APGAR of ≥7), and multiple births provided basic information about newborns. Postnatal factors, such as variables known to be associated with brain development in VP infants, included sepsis, retinopathy of prematurity (ROP), neonatal respiratory distress syndrome (NRDS), and bronchopulmonary dysplasia (BPD).

### 2.3. MR imaging acquisition

A 3.0T GE scanner (Discovery MR750 3.0T) and a 32-channel phased-array head coil were used. Each infant was fed 30 min before the MRI, fitted with silicone earplugs to block scanner noise, and wrapped in a blanket and vacuum immobilization device to promote natural sleep. Neonates who could not remain still were sedated with 10% chloral hydrate (dosage: 25–50 mg/kg) given orally to reduce head movements during the examination. Given the potential risks of chloral hydrate, patient selection, monitoring, and management were strictly performed according to established guidelines. Adverse drug reactions were monitored for 24 h after sedation. The MRI protocol included conventional MRI imaging, 3D T1w imaging, and DTI, and all scans were performed under the supervision of a neonatologist. MRI data were acquired as follows: 3D T1w Bravo: TR = 7.0 ms, TE = 2.6 ms, flip angle = 12°, acquisition plane = sagittal, FOV = 180 mm × 180 mm, acquired matrix = 180 × 180, slice thickness = 1 mm, gap = 0, slice number = 176, acquisition time = 2 min 52 s; DTI data: TR = 3,736 ms, TE = 93 ms, FOV = 180 mm × 180 mm, acquired matrix = 90 × 90, slice thickness = 3 mm, gap = 0, slice number = 32, acquisition time = 6 min 14 s, and *b*-values ranging from 0 to 1,000 s/mm^2^ and 32 non-collinear diffusion-encoding directions.

### 2.4. Diffusion processing

Diffusion tensor imaging images were preprocessed and analyzed using the FMRIB diffusion toolbox (FDT) from the FMRIB software library (FSL; http://www.fmrib.ox.ac.uk/fsl) (Smith et al., 2004). Preprocessing steps included motion and eddy current corrections using the “eddy_correct” tool and gradient correction. The FA and MD images were then calculated by fitting a tensor model to the corrected diffusion data. Whole brain fibers were tracked using a deterministic tracking method. Finally, the FA and MD images were aligned to the newborn template image in the Montreal Neurological Institute (MNI) space using the nonlinear registration tool FNIRT (Rueckert et al., 1999).

### 2.5. Tract-based spatial statistics

Next, mean FA and mean MD images were created and thinned to create a mean FA skeleton and a mean MD skeleton separately, with a threshold of FA > 0.2 or MD > 0.2. The aligned FA and MD images of each subject were then projected onto their respective skeletons, and the resulting data were fed into voxel-wise cross-subject statistics. Statistical analysis was performed using the threshold-free cluster-enhancement (TFCE) method in FSL (Smith and Nichols, 2009). The “randomize” is a permutation test, and 5,000 permutations were performed to fit the group model. Regions with significant FA changes were determined based on a voxel-level height threshold of *p* of <0.03 (uncorrected). Finally, a voxel-wise correlation between the FA and GMFCS levels was performed.

### 2.6. Brain structure connectivity group

The connection network was constructed using the “Pipeline for Analyzing braiN Diffusion imAges” (PANDA) toolbox based on FSL. The FA images were initially aligned with their corresponding T1 images. Then, the anatomical T1 images were standardized in MNI space, and the transformation parameters were inversely transformed and stored. Furthermore, an inverse transformation matrix was used to warp the automated anatomical labeling (AAL) atlas into the individual space for each subject. The AAL atlas of each individual was defined as a node of the structural network. To construct this network, fibers were tracked between each pair of nodes, and the fiber bundle numbers between them were defined as edges. These connections were statistically analyzed using network-based statistics (NBS) in the Gretna software (http://www.nitrc.org/projects/gretna/). A significant change in connections was generated at a *p*-value of < 0.05. The network connectivity graph and the connectivity matrix between the two groups were displayed using the BrainNet Viewer toolkit.

### 2.7. Statistical analysis

SPSS 26.0 was used for statistical calculations. A Student's *t*-test or χ^2^ analysis was used to compare clinical variables between the groups (VP vs. FT) as well as structural network metrics, including global efficiency, local efficiency, small-worldness, and fiber bundle numbers. Statistical parametric mapping (SPM) was used to analyze the correlation between perinatal factors of the VP group and different fiber bundle areas on the TBSS. Regional connectome analyses of group-wise differences (VP vs. FT) were conducted using NBS. Stepwise multiple linear regression analysis was used to study the influence of perinatal factors on the metrics of brain structural networks in the VP group. Through univariate analysis, perinatal factors with a *p*-value of < 0.1 were included in the linear stepwise analysis. A *p*-value of < 0.05 was considered statistically significant.

## 3. Results

### 3.1. Clinical characteristics

Of the 120 infants, 83 were included in this study. A total of 37 infants were excluded: 10 preterm infants at 33–36 weeks GA, 11 with PMA of > 43 weeks on an MRI scan, 5 with visible MRI artifacts, and 11 with congenital malformations or brain injuries, which ultimately included 43 VP and 40 FT infants. There was a significant difference in GA and birth weight between the two groups (*p* < 0.001). Perinatal factors, such as BPD, the APGAR score, intrauterine growth retardation (IUGR), and gestational hypertension, differed significantly between the two groups (*p* < 0.05). The clinical characteristics of the included infants and the distribution of the comorbidities are presented in Table 1.

**Table 1:** Clinical variables in the study population.

**Variables**	**Preterm group (*n* = 43)**	**Term group (*n* = 40)**	***p*-value**
GA at birth (weeks), mean (SD)	27.74 (2.20)	39.38 (1.06)	<0.001
GA at MRI (weeks), mean (SD)	40.96 (1.99)	40.51 (1.11)	0.406
Birth weight (grams), mean (SD)	1045.00 (559.66)	3420.38 (600.88)	<0.001
Men, *n* (%)	23 (53.5)	18 (45.0)	0.440
Multiple births, *n* (%)	18 (41.9)	6 (15.0)	0.007
Proportion of infants with BPD, *n* (%)	12 (27.9)	2 (5.0)	0.013
Infection, *n* (%)	11 (25.6)	3 (7.5)	0.057
5-min APGAR <5, *n* (%)	10 (23.3)	0	0.001
Cesarean section, *n* (%)	16 (37.2)	10 (25.0)	0.231
IUGR, *n* (%)	8 (18.6)	0	0.006
Mother with less than high school education, *n* (%)	8 (18.6)	6 (15.0)	0.661
Respiratory distress at birth, *n* (%)	10 (23.3)	3 (7.5)	0.095
Culture-proven sepsis, *n* (%)	4 (9.3)	2 (5.0)	0.740
Hypertension during pregnancy, *n* (%)	10 (23.3)	2 (5.0)	0.040
Gestational diabetes mellitus, *n* (%)	7 (16.3)	5 (12.5)	0.625
Premature rupture of membranes, *n* (%)	10 (23.3)	6 (15.0)	0.341

Data are presented as mean ± standard deviation (SD) or number (%). Continuous data are analyzed by the t-test. Categorical data are analyzed by the chi-squared test. GA, gestational age; BPD, bronchopulmonary dysplasia; APGAR, activity, pulse, grimace, appearance, respiration; IUGR, intrauterine growth retardation. A p-value of <0.05 indicates a statistically significant difference.

### 3.2. Comparison of white matter microstructure between VP and FT infants

**Figure 1:**
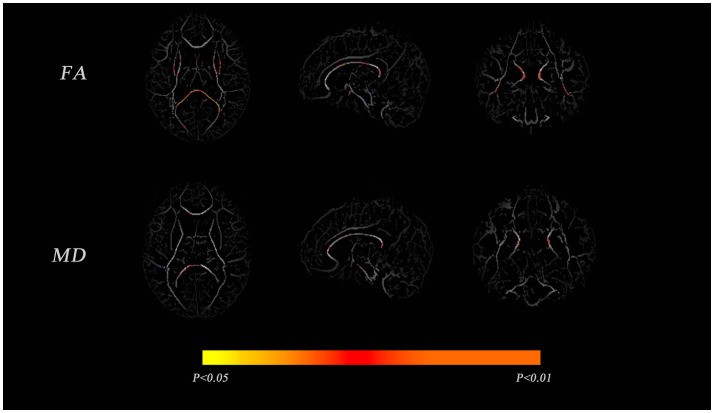
Comparisons of the mean fractional anisotropy (FA) and mean diffusivity (MD) maps between very preterm (VP) and full-term (FT) infants. The mean FA and MD skeletons are shown in gray, and areas with significant differences between the groups are shown in red-yellow (*p* < 0.05).

Tract-based spatial statistics analysis of VP and FT infants showed significant differences in FA between the two groups for the body and splenium parts of the corpus callosum, the anterior limb of the internal capsule, the posterior limb of the internal capsule, and the cingulate gyrus (*p* < 0.01). Only the corpus callosum was significantly different in MD (*p* < 0.01), as shown in Figure 1.

### 3.3. Relationship between white matter FA in the brain and perinatal factors in VP infants

**Figure 2:**
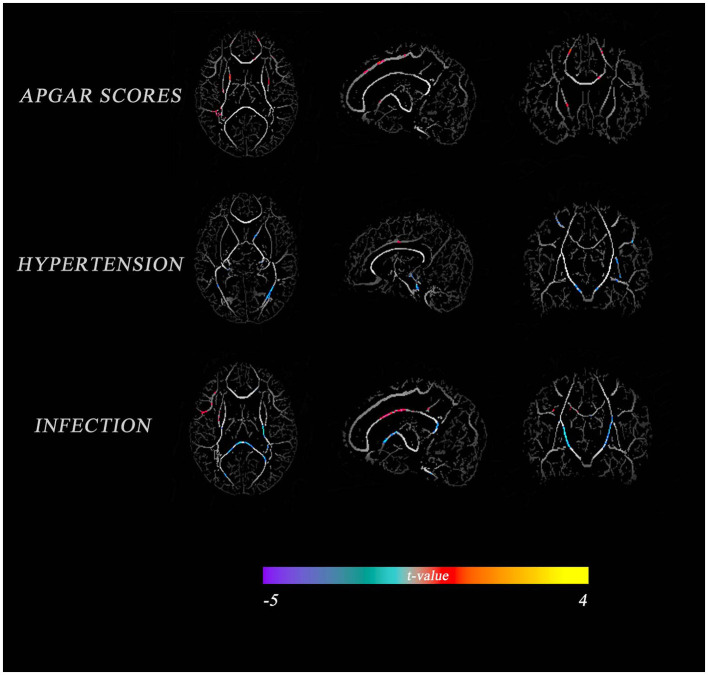
Comparison of perinatal factors between VP and FT infants with the average FA diagram of white matter tracts. The color indicates the *t*-value. Warm tones represent a high correlation, while cold tones represent a low correlation.

A correlation analysis of perinatal factors and TBSS outcomes in VP infants showed that APGAR score, gestational hypertension, and infection were significantly associated with FA in most regions (*p* < 0.05, Figure 2); BPD, multiple births, gender, premature rupture of membranes, GA at birth, and sepsis had small areas correlated with FA (results not shown). In addition, no correlations with FA were observed in some underlying characteristics (i.e., maternal age, education level, birth weight, and invasive ventilation). The APGAR score had a greater positive correlation with FA in the corpus callosum, anterior thalamic radiation, and posterior radiating corona; gestational hypertension had a greater positive correlation with FA in the anterior limb of the internal capsule and optic radiation, and a greater negative correlation with FA in the cingulate gyrus. On the other hand, an infection had a greater positive correlation with FA in the cingulate gyrus and a greater negative correlation with FA in the optic radiation, the corpus callosum superior longitudinal tract, and the FA of the superior longitudinal fasciculus.

### 3.4. Comparison of brain network metrics between VP and FT infants

**Figure 3:**
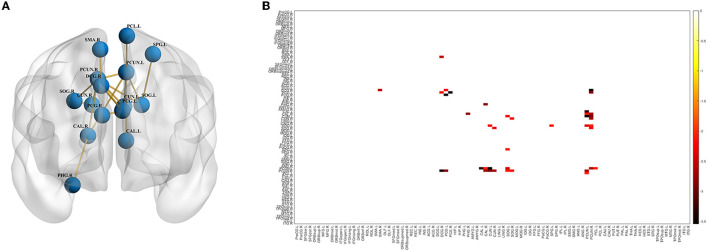
Differences in the brain network between the VP and FT groups. Connection graph **(A)** and connection matrix **(B)** of the preterm and FT groups.

**Figure 4:**
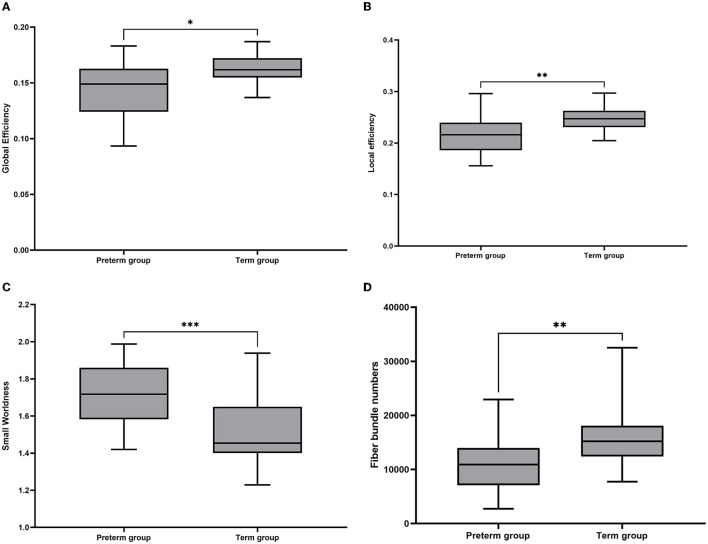
Box plots of overall brain structural network metrics in VP and FT controls. **(A)** Global efficiency; **(B)** local efficiency; **(C)** small-worldness; and **(D)** fiber number. In these plots, the lower and upper edges of each box represent quartiles (i.e., the 25th and 75th percentiles, respectively). Whiskers represent the maximum and minimum. The line inside the box represents the median. *p < 0.05, **p < 0.01, ***p < 0.001.

In the NBS analysis, the mean network differences between the VP and FT groups were compared. There were differences in the strength of connections within the subnetwork involving 15 nodes in the bilateral supplementary motor area, supraoccipital gyrus, precuneus, central gyrus, posterior cingulate gyrus, parahippocampal gyrus, and premotor area (*p* < 0.05) (Figures 3A, B). Of the 15 nodes and 17 connections, six connections were located in the left hemisphere, and five connections in the central node of this network were located in the right precuneus gyrus. Whole-brain network metrics between the VP and FT groups were compared. The VP group had a significantly lower global efficiency (0.1433 ± 0.006186 and 0.1620 ± 0.002400, *p* = 0.0104) and a significantly higher small-worldness (1.7260 ± 0.04104 and 1.5210 ± 0.03553, *p* = 0.0006) than the FT group (Figures 4A, C). The local efficiency of the VP group was also lower than that of the FT group (0.2162 ± 0.03634 and 0.2466 ± 0.02528, *p* = 0.0024) (Figure 4B), but the FT group had more fiber bundle numbers than the VP group (16,193 ± 1,136 and 11,129 ± 1,307, *p* = 0.0063) (Figure 4D).

### 3.5. Relationship between brain network metrics and perinatal factors in VP and FT infants

**Figure 5:**
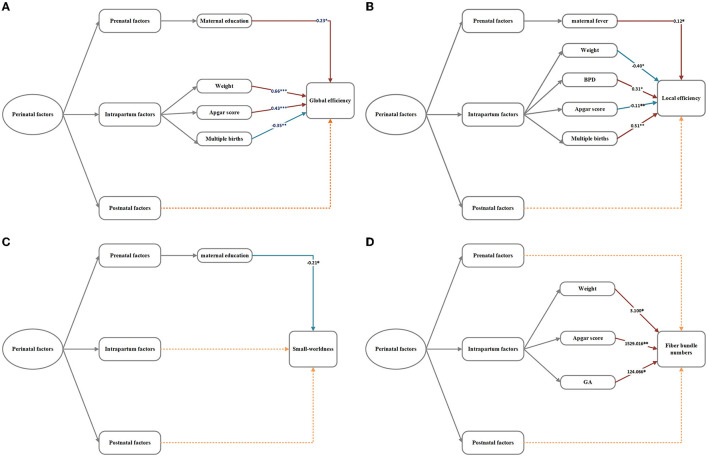
**(A–D)** Regression analysis of network metrics and perinatal factors in the VP group. The numbers in the model represent the correlation coefficient *b*-values. *p < 0.05, **p < 0.01, ***p < 0.001. The dotted lines signify insignificant relationships.

In multiple linear regression analyses of all perinatal factors and network metrics (Figure 5), low birth weight was associated with decreased global efficiency, increased local efficiency, and decreased number of fiber bundles. Higher APGAR scores were associated with increased global efficiency, decreased local efficiency, and increased number of fiber bundles but not small-worldness. Multiple births were associated with decreased global efficiency and increased local efficiency. Higher maternal years of education were associated with increased global efficiency and decreased small-worldness. A higher fever rate and BPD were only associated with increased local efficiency, and a higher GA was associated with increased fiber bundle numbers.

## 4. Discussion

### 4.1. Analysis of the differences in the different nerve fiber bundles between preterm and term infants, and the clinical significance of the underlying pathology

Compared with FT infants, most white matter tracts of VP infants were delayed at TEA. The differences in fiber bundles between VP and FT infants were in the corpus callosum, corticospinal tract, and visual radiation, which controlled cognitive, motor, and visual functions, respectively. This finding was basically consistent with the delay area obtained in most previous studies (Pannek et al., 2014; Jin et al., 2019). Moreover, the corpus callosum differed mainly from the FT group in the body and splenium, with little difference in the mouth and the genu. This might be related to the growth sequence of the corpus callosum: mouth-genu-body-splenium (Rademaker et al., 2004; Ren et al., 2006). The corpus callosum is mainly a fibrous plate of fibers linking the left and right hemispheres of the brain, which maintains the coordination of activities between the two hemispheres. Fibers passing through the genu connect the left and right frontal lobes, and those passing through the body connect the rear and parietal lobes. Fibers passing through the splenium connect the left and right temporal lobes, parietal lobes, and occipital lobes, indicating that the genu of the corpus callosum is closely related to the function of the frontal lobes, the body is closely related to the frontal and parietal lobes, and the splenium is closely related to the temporal, parietal, and occipital lobes. As a result, VP infants have lower motor, sensory, cognitive, and visual abilities than FT infants at TEA.

### 4.2. Analysis of differences in the structural brain network between VP and term infants and the pathological clinical significance behind them

This study found that the fiber bundle numbers in the VP group were significantly different from those in the FT group. The smaller fiber bundle numbers in the VP group compared to the FT group suggested that the average strength of connections in the brain network was weaker in VP infants than in FT infants. Fiber bundle numbers could determine the average strength of connections in the brain network because the fiber bundle numbers connecting the two cortical nodes were first normalized to the node volume (the sum of the two nodes) and then normalized to the sum of fiber bundle numbers in the whole brain (Liu et al., 2022). The smaller the fiber bundle numbers and the weaker the average connection strength, the less dense the network. The remaining network metrics, such as global efficiency, local efficiency, and small-worldness, depend on the network density (Rademaker et al., 2004). The global and local efficiencies of the whole brain structural networks were lower in VP than in FT infants. It was suggested that VP infants had less ability to integrate and separate networks than FT infants. This may be due to the fact that VP infants spent less time in the mother's body than FT infants and therefore lacked some of the processes required for the proper maternal environment and neurodevelopment than FT infants. Preterm birth can affect and disrupt the local and global efficiency of the network. The ability to integrate rapidly specialized information from distributed nodes was negatively affected by preterm birth and was accompanied by a decline in global efficiency. VP infants had an overall less efficient network than FT infants, which might lead to a later delay or interruption of brain development (Thompson et al., 2015). Our study also demonstrated that both VP and FT infants have small-worldness in their brain's structural networks and that small-world networks were intermediate between the random and regular networks (Brown et al., 2014). The random network has a smaller level of cluster coefficients, while the regular network has larger cluster coefficients. The construction of small-world networks in VP infants was intended to strike an optimal balance between functional integration and separation. VP infants have increased small-worldness, inefficient local information transfer in the brain, and fragmented modular structures, suggesting delayed or abnormal development of the ability to integrate information between brain regions (Bouyssi-Kobar et al., 2019). Interestingly, we also found that VP infants had lower connectivity than FT infants in 15 nodal brain regions, including the bilateral supplementary motor area, supraoccipital gyrus, precuneus, central gyrus, posterior cingulate gyrus, parahippocampal gyrus, and premotor area. These areas were intrahemispherically and interhemispherically distributed. The central node of this network was the right precuneus gyrus. The precuneus was associated with many high-level cognitive functions such as situational memory, self-related information processing, and aspects of consciousness. It involves anatomical cortico–subcortical and cortico–cortical connections, including connections to the inferior parietal and superior temporal regions. It is known from structural and functional connectivity research, to be an important hub and related to the default pattern network (Cavanna and Trimble, 2006; Gong et al., 2009). In the left hemisphere, we found that the connections between the anterior cuneiform gyrus and the posterior cingulate gyrus were reduced in VP infants, which might be related to a reduced ability to process and distribute information to the outside world. In addition, in the present study, brain regions with reduced connectivity in VP infants were mainly located in the parietal and occipital regions. This might be related to the developmental sequence of the brain. VP infants had less developed parietal and occipital lobes than FT infants due to the early onset of life outside the womb, and these infants had lower network connectivity than FT infants. It was not difficult to explain the speech and visual impairments that occurred in VP infants during their later development.

### 4.3. Analysis of the correlation between perinatal factors and abnormalities in brain fiber tracts and structural networks

In addition, this study described some perinatal factors associated with white matter tracts and structural connectivity in VP infants. Factors associated with white matter microstructure included the APGAR score, hypertension, and infection. Gestational hypertension affected the development of white matter microstructure in infants, and many studies supported this view (Xing et al., 2021; Zheng et al., 2022). As the severity of gestational hypertension increased, the degree and extent of brain damage in preterm infants gradually increased, as did the inflammatory response. Inflammatory mediators inhibit the differentiation and maturation of oligodendrocyte precursors, cause myelin degradation, soften the cerebral white matter, and alter the FA and MD values in white matter tracts. High APGAR scores were associated with elevated FA values in white matter tracts. The criterion evaluated in this study was an APGAR score of <7 because that was the threshold value that distinguishes hypoxia from normality. Higher APGAR scores were not only associated with white matter microstructure but also with increased global efficiency of the structural network, increased fiber bundle numbers, and decreased local efficiency. Although few reports have shown that the infection can cause changes in white matter tracts, the current study has found that the infection was associated with lower FA values in white matter tracts, and the main relevant white matter tract region was the optic radiation. Because infection caused the onset of prematurity and slowed the development of brain fiber tracts in preterm infants. Factors associated with structural connectivity included maternal years of education, infant weight, APGAR score, multiple births, GA at birth, maternal fever, and BPD. Low birth weight, multiple births, and younger GA at birth have been reported to be associated with poorer connectivity strength (Thompson et al., 2016). Our study demonstrated that low birth weight and multiple births were associated with a decrease in global efficiency and an increase in local efficiency. Maternal fever was also associated with an increase in local efficiency. The increased local efficiency of these preterm infants might reflect a compensatory mechanism that increased the local efficiency of VP infants due to physiological and pathological changes that resulted in increased stress resilience. To some extent, this reflected the ability of the brain networks of VP infants to resist random attacks. Previous studies have demonstrated that lung disease can affect white matter microstructure in VP infants (Ball et al., 2010). We found evidence of an association between bronchopulmonary dysplasia and poor network connectivity. Several previous studies have demonstrated that white matter microstructure and brain networks can be influenced by gender as a factor (Schmidbauer et al., 2022). Although the perinatal factors in this study were more comprehensive, no effect of sex on white matter microstructure and brain network connectivity was found, probably due to the uneven distribution of sex in our subgroups. Surprisingly, we found evidence of an association between maternal years of education and decreased small-worldness and increased global efficiency in VP infants. Surprisingly, we found an association between higher years of maternal education and increased global efficiency. In China, mothers with more years of education were more knowledgeable about fetal care and how to reduce the risk of preterm birth. They would make more preparations for pregnancy, such as taking folic acid and having regular maternity visits. They also got rid of bad lifestyle habits like smoking and drinking coffee or alcohol. This is the first study to report the relationship between higher maternal years of education and structural connectivity in VP infants. Nevertheless, previous studies did not mention that years of maternal education affected the network of VP infants (Thompson et al., 2019). This may be due to the differences in data on Chinese infants. Different educational backgrounds in different social states may yield different outcomes.

A major strength of this study is the combination of brain MRI and perinatal factors. TBSS was used to calculate voxel differences between individuals in FA and MD (Kelly et al., 2020). In contrast, a network of differences between individuals was constructed using structural networks in addition to TBSS, and a correlation analysis was performed between clinical factors and metrics of the different fiber bundles or brain regions. The combination of both methods made perinatal factors more convincing. Next, while previous studies were based on data from European and American infants, our study looked at brain development in China's preterm infants. Finally, perinatal factors were divided into three levels: prenatal, intrapartum, and postnatal. This made perinatal factors more comprehensive and structured.

Nevertheless, this study had some limitations. First, head artifacts were considered to be an unavoidable factor in infant MRI. Several head artifacts that can affect images continue to affect the construction of fibers and networks. Second, the TBSS technique is sensitive to noise, and subsequent studies should consider a better alternative method. Third, we were unable to subdivide the preterm birth group according to the GA range because of sample size limitations. The results might be affected by uneven GA, and the sample size should be expanded in the future to more precisely analyze the effects of perinatal factors on white matter microstructure and brain network connectivity in premature infants of varying degrees. Additionally, we did not use the follow-up results to verify how the network and white matter microstructure changed as preterm infants developed. Further research will be done in the following areas.

## 5. Conclusion

This study has improved our understanding of perinatal factors that may alter brain fiber bundles and structural network connections in infants. These changes may improve our understanding of the mechanisms of brain injury in Chinese VP infants and better guide clinical intervention and treatment.

## Data availability statement

The original contributions presented in the study are included in the article/supplementary material, further inquiries can be directed to the corresponding authors.

## Ethics statement

The studies involving human participants were reviewed and approved by the Third Medical Center of Chinese PLA General Hospital. Written informed consent to participate in this study was provided by the participants' legal guardian/next of kin. Written informed consent was obtained from the minor(s)' legal guardian/next of kin for the publication of any potentially identifiable images or data included in this article.

## Author contributions

YL contributed to the data processing and manuscript writing. BN contributed to the conception of the study. YW, FH, and QM contributed to MRI scanning. TH, HZ, and JL contributed to the participants recruitment. GM contributed to the analysis of neuroimaging data. XM and BW contributed to the conception and design of the study and major revision of manuscript. All authors contributed to the article and approved the submitted version.
